# Disturbance in the Mucosa-Associated Commensal Bacteria Is Associated with the Exacerbation of Chronic Colitis by Repeated Psychological Stress; Is That the New Target of Probiotics?

**DOI:** 10.1371/journal.pone.0160736

**Published:** 2016-08-08

**Authors:** Sohei Arase, Yohei Watanabe, Hiromi Setoyama, Noriko Nagaoka, Mitsuhisa Kawai, Satoshi Matsumoto

**Affiliations:** Yakult Central Institute, Kunitachi-shi, Tokyo, Japan; Case Western Reserve University, UNITED STATES

## Abstract

Psychological stress can exacerbate inflammatory bowel disease. However, the mechanisms underlying how psychological stress affects gut inflammation remain unclear. Here, we focused on the relationship between changes in the microbial community of mucosa-associated commensal bacteria (MACB) and mucosal immune responses induced by chronic psychological stress in a murine model of ulcerative colitis. Furthermore, we examined the effect of probiotic treatment on exacerbated colitis and MACB composition changes induced by chronic psychological stress. Repeated water avoidance stress (rWAS) in B6-*Tcra*^-/-^ mice severely exacerbated colitis, which was evaluated by both colorectal tissue weight and histological score of colitis. rWAS treatment increased mRNA expression of *UCN2* and *IFN-γ* in large intestinal lamina propria mononuclear cells (LI-LPMC). Interestingly, exacerbated colitis was associated with changes in the microbial community of MACB, specifically loss of bacterial species diversity and an increase in the component ratio of *Clostridium*, revealed by 16S rRNA gene amplicon analysis. Finally, the oral administration of a probiotic *Lactobacillus* strain was protective against the exacerbation of colitis and was associated with a change in the bacterial community of MACB in rWAS-exposed *Tcra*^-/-^ mice. Taken together, these results suggested that loss of species diversity in MACB might play a key role in exacerbated colitis induced by chronic psychological stress. In addition, probiotic treatment may be used as a tool to preserve the diversity of bacterial species in MACB and alleviate gut inflammation induced by psychological stress.

## Introduction

Inflammatory bowel disease (IBD), which includes ulcerative colitis (UC) and Crohn’s disease (CD), is an intractable disease in the modern society [1]. Chronic inflammation associated with IBD follows a course of relapse and remission, and longstanding IBD has been shown to increase the risk of colorectal cancer [2]. Psychological stress is one of the factors that can exacerbate IBD [3]. Exposure to psychological stress has been shown to exacerbate dextran sulfate sodium (DSS)–induced colitis in the murine model [4]. However, it remains unclear how psychological stress affects gut inflammation.

There are many studies suggesting that gastrointestinal (GI) microbiota is involved in the development and exacerbation of chronic colitis [5]. Studies suggested that an increase in *Clostridium difficile* is correlated with severity of colitis [6]. Involvement of GI microbiota has been implicated not only in gut inflammation, but also in colorectal cancer, type 1 diabetes, and obesity [7]. It has been speculated that changes in the gut microbiota alters mucosal permeability, which results in dysfunction of immune responses in the colonic mucosa [8].

Psychological stress is known to affect the GI microbiota [9]. For example, 7 day repeated exposure to water avoidance stress (rWAS) has been shown to alter the composition of colonic microbiota [10]. In addition, other psychological stressors have also been demonstrated to reduce microbial species diversity (dysbiosis) and increase pathogenic bacteria in the murine intestine [11, 12]. However, little is known about how psychological stressors induce changes in GI microbiota, and the mechanism by which altered GI microbiota accelerates gut inflammation. Therefore, we investigated the impact of psychological stress on both gut microbiota and the mucosal immune system in the colon to clarify the factors that intensify gut inflammation in this setting. We utilized a T cell receptor alpha chain gene (*Tcra*) knockout mouse model, which spontaneously develops human UC-like chronic colitis, apart from germ-free conditions [13]. We analyzed the microbiota located in the colonic mucosa (mucosa-associated commensal bacteria: MACB), as studies have suggested that this microbial community may be clinically important in IBD patients [14]. Secondly, we investigated the effect of probiotics on accelerated gut inflammation induced by psychological stress. Probiotics are defined as living microorganisms that confer beneficial health effects to the host, and many studies have previously examined the clinical impacts of probiotics on IBD patients [15, 16]. While several probiotics have demonstrated beneficial effects on IBD, the mechanisms underlying these effects remain unknown. As described above, loss of microbial species diversity in GI microbiota is a typical characteristic in IBD patients. We speculate that the beneficial effects of probiotics may be due to stabilization of the GI microbiota.

## Materials and Methods

### Mice

Female 8 week old *Tcra*^-/-^ mice on the C57BL/6 background (B6.129P2-*Tcra*^*tm1Mom*^/Yit; *B6-Tcra*^*-/-*^) were kindly provided to us by Dr. Susumu Tonegawa (Massachusetts Institute of Technology, Cambridge, MA, USA). Studies indicated that C57BL/6 and BALB/c, which were the available genetic backgrounds for *Tcra*^*-/-*^ mice, show differential stress responses in the central nervous system and the gut [17]. We found that C57BL/6 mice showed higher sensitivity to chronic psychological stress as compared with BALB/c mice (data not shown), and therefore our experiments were performed using the B6-*Tcra*^*-/-*^ mice. Furthermore, we have compared sex differences in the stress response to rWAS stimulation. In this experiment, plasma corticosterone levels and intestinal permeability were higher in female C57BL/6 mice compared to that of male mice (data not shown). It is suggested that female mice have more sensitivity to psychological stress; therefore, we used female mice in our study. Animals were maintained in ventilated cases (containing autoclaved soft bedding, water, and food), in an environmentally controlled room (23 ± 1°C, 12 h light/dark cycle). After the stress session, mice were euthanized by isoflurane inhalation and samples were collected for analysis. All animal experiments were approved by the Institutional Animal Care and Use Committee of Yakult Central Institute (approval number: 13–0091, 14–0130).

### Repeated water avoidance stress (rWAS)

In most previous reports, exposure term of psychological stress in mice was less than 10 days [8, 10–12], and no study has examined the influence of long-term psychological stress on IBD. To elucidate the relationship among chronic psychological stress, GI microbiota, and chronic inflammation, mice were subjected to repeated water avoidance stress (rWAS) for 12 weeks. It was performed between 9:00 am and 2:00 pm for 1 hour per day, and was repeated 5 days per week for 12 weeks (60 times in total). Mice were placed individually on a rectangular platform (3 cm length × 3 cm width × 6 cm height), which was affixed (in the center) to the bottom of a container (40 cm length × 25 cm width × 20 cm height). The container was filled with water up to 1 cm below the top of the platform. It is known that *Tcra*^*-/-*^ mice have individual differences in developing colitis between the age of 8 and 20 weeks. Therefore, we selected a 12 week term of rWAS for this study to clarify the influence of chronic psychological stress on the development of colitis. We also examined two different rWAS protocols, once or five times per week for 12 weeks, to determine the rWAS frequency appropriate for exacerbating colitis. The result indicated that high frequency rWAS (five times per week) induced more severe colitis, with persistent activation of the hypothalamus–pituitary-adrenal (HPA) axis, compared with low frequency rWAS (once a week) (S1 Fig). Therefore we reasoned that high frequency rWAS was suitable for the purpose of this study.

### Administration of *Lactobacillus casei* strain Shirota (LcS)

LcS (YIT9029) is one probiotic strains that can survive in the intestine and modulate GI environment. LcS fermented milk (> 5 billion CFU /ml) and the placebo were placed in sealed packs and set on the breeding cages. Instead of water, mice were given 2 mL of either LcS or placebo between 9:00 am and 2:00 pm 5 days a week, for 12 weeks (same time period as the rWAS sessions). Feces from mice that were given LcS fermented milk were collected once a week, and total LcS was measured by the colony detection method. More than 10^9^ CFU of LcS per gram of feces was detected from each mouse (data not shown).

### Measurement of plasma corticosterone concentration

Blood samples were collected into heparinized tubes from the tail vein at 8 am, before dissection. The samples were centrifuged and plasma was stored at -80°C until measurement. The concentration of plasma corticosterone was measured using a Corticosterone ELISA Kit (Enzo Life Sciences, Farmingdale, NY, USA) according to the manufacturer’s instruction.

### Assessment of disease severity

After 12 weeks of rWAS exposure, mice were euthanized, and the entire large intestine was excised from each mouse. Immediately following the removal of large intestine, a 1 cm segment of the proximal colon was isolated and fixed as frozen tissue sample. The frozen tissue was sectioned by cryostat, mounted on microscope slides, and stained with hematoxylin–eosin (H-E). Remaining colorectal tissue was then washed with 10 mM phosphate-buffered saline (PBS), and the tissue weight was measured. Severity of colitis was determined by both histological scoring of the proximal colon and colorectal tissue weight, as previously described with minor modifications [18]. “Table 1” outlines the method for histological scoring of colitis [19]. Blinded histological examination was performed by a pathologist. Each parameter was given a score ranging from 0 to 3, and total histological score was calculated as the sum of all scored parameters.

**Table 1:** Histopathological scores for the evaluation of colitis in *Tcra*^−/−^mice.

	Score			
Histological changes	0	1	2	3
Active inflammation	None	PMN accumulation (mild)	PMN accumulation (severe) without crypt abscess	PMN accumulation (severe) with crypt abscess
Chronic inflammation	None	MNC accumulation (mild)	MNC accumulation (severe) in LP	MNC accumulation (severe) in LP and SM
Epithelial hyperplasia	None	Mild	Intermediate	Moderate
Ulcer	None	Erosion (mild)	Erosion (severe)	Ulcer
Malignancy	None	LGD	HGD	Invasive cancer
Extension	None	Focal (30%)	60%	Entire region

PMN: polynuclear leucocyte, MNC: mononuclear cell, LP: lamina propria, SM: submucosal, LGD: low-grade dysplasia, HGD: high grade dysplasia. The scores were adapted from the Wallace score [19].

### Flow cytometric analyses of lamina propria mononuclear cells in large intestine (LI-LPMC)

LI-LPMCs were isolated from mice as previously described [20]. LI-LPMCs were stained with mAbs (4 μg/ml; BD Biosciences, San Jose, CA, USA): hamster anti-mouse TCRβ (H57-597) and rat anti-mouse CD4 (RM4-5), and analyzed with the cell-analyzer (Gallios^™^; Beckman Coulter, Inc., Brea, CA, USA).

### Reverse-transcription polymerase chain reaction

Total LI-LPMC mRNA was purified from LI-LPMCs using the RNeasy Mini Kit (QIAGEN, Venlo, Netherlands). Reverse transcription was carried out with 1 μg of RNA using 200 U of Superscript II RNase H- RT (Invitrogen^™^, Carlsbad, CA, USA). Complementary DNA (cDNA) was amplified by polymerase chain reaction. In this analysis, chronic inflammatory responses in LI-LPMC were evaluated by the gene expression of *IFN-γ*, *IL-6*, and *TNF-α*. The expression of other inflammatory cytokines such as *IL-1β*, *IL-10*, *IL-17α*, *IL-18*, *IL-22*, and *IL-23* was also measured, but did not change (S1 Table). Urocortins (UCNs) belong to the corticotropin releasing hormone (CRH) family, and are known to bind CRH receptors (CRHRs) to activate CRH-mediated pathways in peripheral tissues [21]. An increase in CRH is a known indicator of activation of the central nervous system (CNS) [22], and we considered UCNs to be an indicator of this in peripheral tissue. Expression of all genes in each sample was quantified relative to glyceraldehyde 3-phosphate dehydrogenase (*GAPDH*).

### Bacterial 16S rRNA gene amplicon analysis (16S amplicon analysis) in mucosa-associated commensal bacteria (MACB)

Frozen sections of proximal colon were mounted on membrane slides (PALM membrane slide, Carl-Zeiss, Jena, Germany). The upper area of the colonic epithelium (approximately 20 μm) was defined as the mucosal region, which we collected by Laser capture Micro Dissection (P.A.L.M. Laser Beam IV., Carl-Zeiss, Jena, Germany) into adhesive caps (Carl-Zeiss, Jena, Germany). DNA of MACB within membrane samples was extracted using the QIAmp DNA micro kit (QIAGEN, Venlo, Netherlands).

Amplification and sequencing of the V4 region within the bacterial 16S rRNA gene was performed on extracted DNA from the mucosal region using the primers 515F and 806R, as previously described with minor modifications [23]. Barcoded amplicons were obtained using SYBR premix Extaq (Takara Bio Inc., Shiga, Japan), and the reaction was carried out in a final volume of 50 μl, with 10 ng template rDNA. The PCR amplification program was as follows: one cycle at 95°C for 10 s, then 30 cycles of 95°C for 5 s and 55°C for 40 s. Amplicons were purified with the High Pure PCR product Purification kit (Roche Diagnostics GmbH, Mannheim, Germany), and were quantified using the Quant-iT PicoGreen dsDNA Assay Kit (Invitrogen^™^, Carlsbad, CA, USA). Finally, equimolar concentrations of each sample were pooled and sequenced on the Illumina MiSeq platform using the MiSeq Reagent Kit v2 (Illumina, Inc., San Diego, CA, USA).

### Bioinformatics analysis

The sequences acquired from Miseq were analyzed using the Quantitative Insights Into Microbial Ecology (QIIME) software package version 1.8.0 [24]. All protocols were performed as previously reported [25]. Raw 250-bp paired-end sequence reads were combined, and quality filtering was performed [26, 27]. The chimeras in the sequence reads were detected and removed using the USEARCH and UCHIME algorithms [28, 29]. The 16S rRNA operational taxonomic units (OTUs) from the combined reads that clustered at 97% were collected, and the selected OTUs were assigned to taxa and aligned [30–32]. The most abundant sequence in each OTU was extracted as a representative, and analyzed by local BLAST [33]. Relative abundance of bacterial taxa was computed and collated. Sequence libraries were randomly sub-sampled to achieve even sampling depth (20,000 reads per sample) before diversity metrics were calculated. The OTU diversity within and between samples was compared using α and β diversity indices, respectively. α diversity was measured with the observed species metrics. β diversity was evaluated using the weighted UniFrac pipeline in QIIME [34]. We then tested for differences with the methods implemented in QIIME (make_distance_boxplots.py script with the nonparametric options) for comparisons based on UniFrac distance. Principal Coordinate Analysis (PCoA) was used to interpret and visualize the variations in weighted UniFrac distance matrix. The largest amount of variation was explained by the first principal coordinate (PCo1), and the second largest by the second principal coordinates (PCo2). Significant variations in frequency of genera and OTUs across groups were extracted by the group_significance.py script. sequence data were deposited in the DDBJ Sequence Read Archive (DRA) under the BioProject Accession No. PRJDB4314.

### Measurement of IgA and IgG concentration in colonic contents

Colonic contents were diluted 10-fold with 10 mM phosphate-buffered saline (pH 7) and centrifuged. The concentrations of IgA and IgG in the serially diluted supernatants were measured using ELISA, as previously described [35].

### Statistical analysis

Data are presented as mean ± SE. Significant variations in frequency of genera and OTUs across groups were extracted and nonparametric t-tests with the Benjamini-Hochberg’s False Discovery Rate (FDR) correction was used. Other comparisons between 2 groups were evaluated using the unpaired Student’s *t*-test. Probability values of less than 5% were considered significant.

## Results

### Involvement of rWAS in exacerbation in gut inflammation

**Fig 1:**
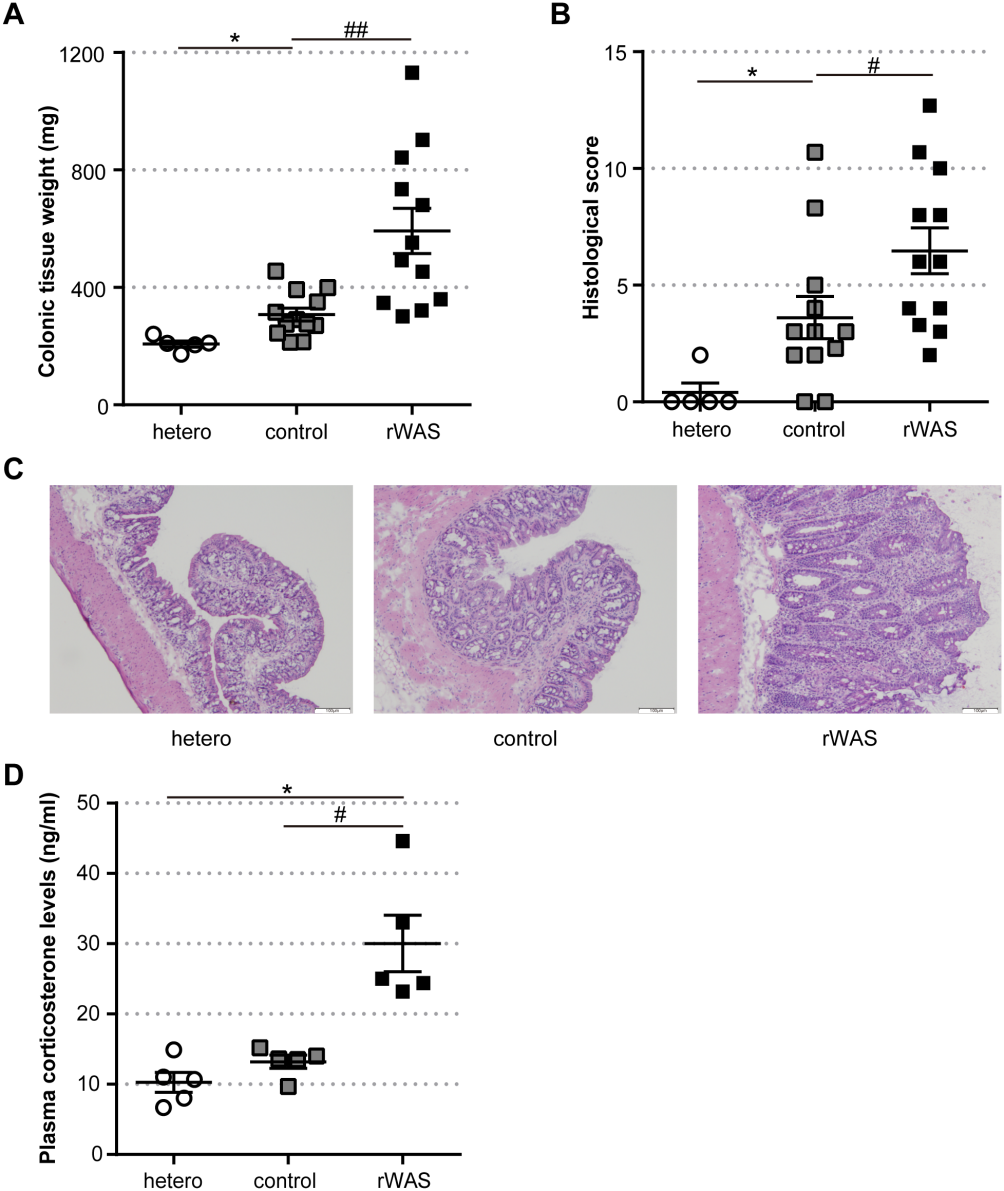
Exacerbation of colitis induced by rWAS in *Tcra*^−/−^ mice. (A) Colonic tissue weight. (B) Histological scores of colitis. Each point represents an individual animal; horizontal bar with errors represents mean ± SE, n = 5: hetero group, n = 12: control and rWAS group. (C) Representative colonic tissue of hetero, control or rWAS mice with H-E staining. Scale bars indicate 100 μm. (D) Corticosterone concentration in the serum. Data are presented as mean ± SE, n = 5 per group. hetero: *Tcra*^−/+^ mice not exposed to repeated water avoidance stress (rWAS), control: *Tcra*^−/−^ mice not exposed to rWAS, rWAS: *Tcra*^−/−^ mice exposed to rWAS. Data were pooled from 2 independent experiments. * p < 0.05 (hetero vs. control), ^#^ p < 0.05, ^##^ p < 0.01 (control vs. rWAS).

To investigate the effect of rWAS on gut inflammation in *Tcra*^-/-^ mice, colorectal tissue was excised from *Tcra*^-/-^ mice with or without rWAS exposure, and disease severity was evaluated via colorectal tissue weight and histological score of colitis. Results indicated that both colorectal tissue weight and colitis score in rWAS-exposed *Tcra*^-/-^ mice were significantly increased as compared with those of control *Tcra*^-/-^ mice (Fig 1A and 1B). Activation of the HPA axis was evaluated through the corticosterone concentration in the serum. The data indicated that the constitutive concentration of plasma corticosterone was increased by rWAS exposure (Fig 1D). To evaluate the mucosal immune responses in colonic mucosa following rWAS exposure, the cell number in inflammatory infiltrates was quantified by flow cytometry. And, gene expression of inflammatory cytokines (*IFN-γ*, *IL-6*, and *TNF-α*) and peripheral stress-associated peptides (*UCN2* and *CRHR2*) in LI-LPMCs was assessed by quantitative RT-PCR. The cell number of CD4^+^ TCRβ^dim^ T cells in LI-LPMCs of rWAS-exposed *Tcra*^-/-^ mice was increased as compared with that of control *Tcra*^-/-^ mice (Table 2), which was consistent with previous reports. Furthermore, myeloperoxidase activity in feces was increased in *Tcra*^-/-^ mice exposed to rWAS and this finding was indicative of accumulation of neutrophils in the colonic mucosa of *Tcra*^-/-^ mice exposed rWAS (S1 Table). Exposure to rWAS also induced the increase of gene expression of *IFN-γ* in LI-LPMCs. Additionally, we found that gene expression of *UCN2* and *CRHR2*, were significantly increased in LI-LPMCs of mice subjected to rWAS (Table 2). Interestingly, the mRNA levels of other cytokines, including *IL-1β*, *IL-10*, *IL-17α*, *IL-18*, *IL-22*, and *IL-23*, in LI-LPMCs did not change (S1 Table). Furthermore, we examined the effects of rWAS on physiological conditions in C57BL/6 mice. rWAS exposure increased the gene expression levels of *IFN-γ*, *UCN2* and *CRHR2* in LI-LPMCs (S2 Table); however, there was no sign of colitis in C57BL/6 mice (S2A Fig).

**Table 2:** Cell number of CD4^+^ TCRβ^dim^ cells and mRNA expression of LI-LPMC cytokines.

	control	rWAS
Cell numbers (×10^4^ cells)		
CD4^+^ TCRβ^dim^	3.0 ± 0.9	46.0 ± 22.0
Gene expression levels of mRNA		
*UCN2*	2.1 ± 0.7	10.7 ± 3.2*
*CRHR2*	2.1 ± 0.8	13.5 ± 3.8*
*IFN-γ*	3.5 ± 1.2	14.1 ± 4.0*
*IL-6*	5.2 ± 2.6	3.8 ± 0.3
*TNF-α*	1.1 ± 0.1	0.9 ± 0.1

Cell number of CD4^+^ TCRβ^dim^ cells was calculated by multiplying the percentage of CD4^+^ TCRβ^dim^ cells (via flow-cytometry) by the number of mononuclear cells (determined via microscopy). Gene expression of mRNA in LI-LPMC was determined by quantitative RT-PCR with ABI-7500. control: *Tcra*^−/−^ mice not exposed to repeated water avoidance stress (rWAS), rWAS: *Tcra*^−/−^ mice exposed to rWAS. Data are presented as mean ± SE (n = 8: control group, n = 12: rWAS group). Data were pooled from 2 independent experiments.

* p < 0.05.

### Changes in bacterial species diversity and composition of mucosal-flora by rWAS

**Fig 2:**
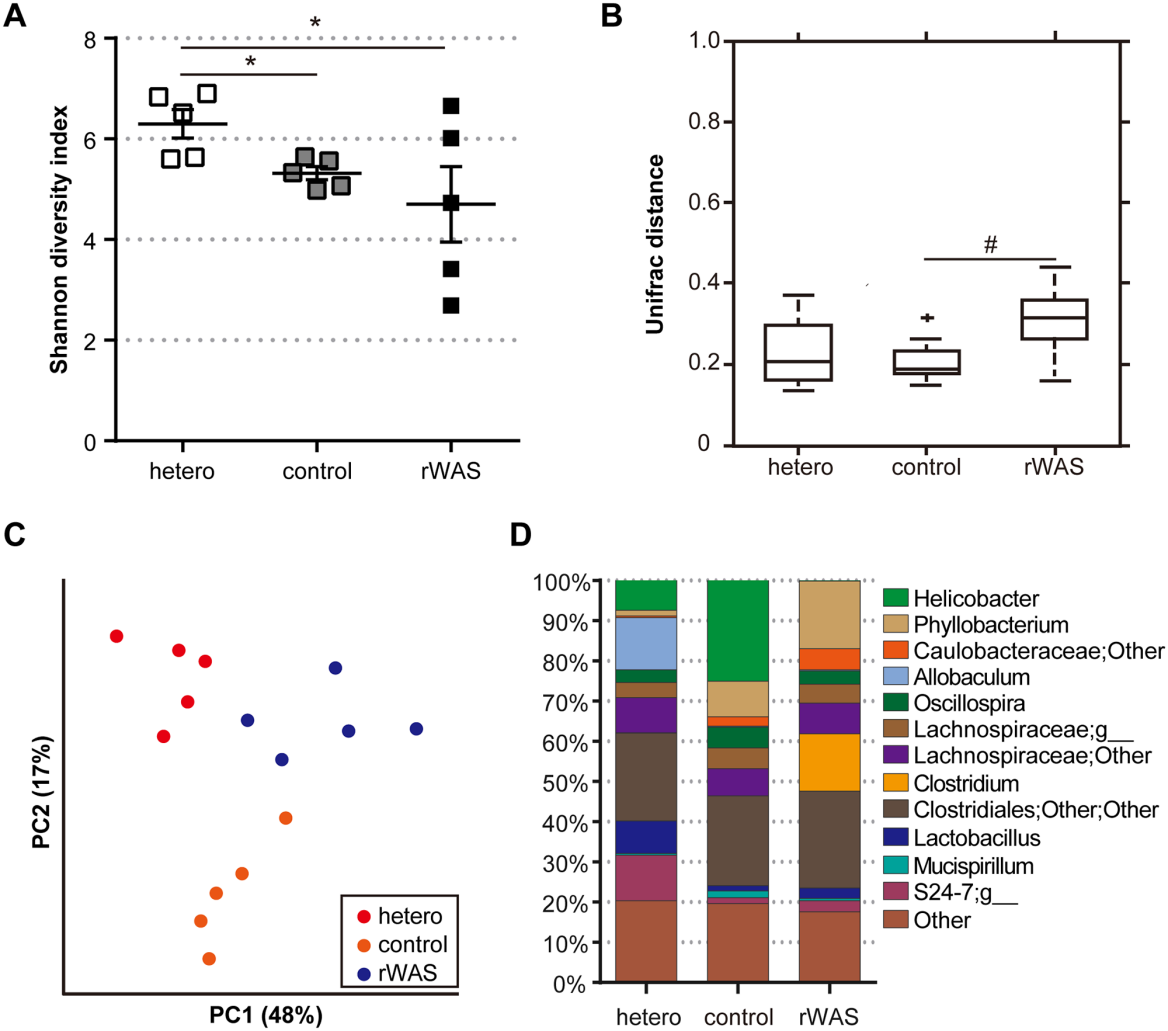
Effect of rWAS on microbial species diversity and genus composition in MACB of *Tcra*^-/-^ mice. (A) Shannon diversity index. Each point represents an individual animal; horizontal bar with errors represents mean ± SE, n = 5 per group. (B) Group distance. Data are presented as box plots, n = 5 per group. (C) Pcoa plot. Each point represents an individual animal, n = 5 per group. (D) Composition of MACB at the genus level. Data are presented as staked bar chart, n = 5 per group. hetero: *Tcra*^−/+^ mice not exposed to repeated water avoidance stress (rWAS), control: *Tcra*^−/−^ mice not exposed to rWAS, rWAS: *Tcra*^−/−^ mice exposed to rWAS. * p < 0.05 (hetero vs. control, hetero vs. rWAS), ^#^ p < 0.05 (control vs. rWAS).

We investigated the impact of rWAS exposure on the bacterial communities of the colonic mucosa via analysis of the 16S amplicon. Species richness was found to be significantly lower in the MACB of rWAS-exposed *Tcra*^-/-^ and control mice as compared with that of hetero mice (Fig 2A). Although the difference between control and rWAS-exposed *Tcra*^*-/-*^ mice was not statistically significant, the diversity in the MACB was significantly correlated with the severity of colitis in rWAS-exposed *Tcra*^*-/-*^ mice (R = -0.68, P = 0.03). The weighted UniFrac distance in the MACB of rWAS exposed *Tcra*^-/-^ mice was significantly larger than that of control mice (Fig 2B). Principal-coordinates analysis (pcoa) plot also showed that the MACB cluster of rWAS exposed *Tcra*^-/-^ mice was more distant as compared with that of control mice (Fig 2C). Comparing the composition of microbial communities in MACB, unclassified genera of the S24-7 family and the *Allobaculum* genus decreased, and the *Phyllobacterium* genus increased with *Tcra* deficiency (both control and rWAS mice (FDR-corrected *P* value = 0.04), Fig 2D). Abundance of the *Helicobacter* genus was not statistically significant and not correlated with the severity of colitis (R = -0.01). Following exposure to rWAS, OTUs were significantly increased in *Clostridium* (*P =* 0.04). Focusing on the detection ratio and relative abundance of OTUs that significantly changed in MACB of rWAS-treated mice, increases in OTUs 4445673, 4481624 and 4409730, and decrease in OTU 184451 became apparent, which showed high similarity with *Clostridium perfringens*, *Clostridium dispolicum*, *Clostridium bifermentans*, and *Clostridium scindens*, respectively (Table 3). Notably, the component ratio of *Clostridium perfringens* was correlated with the severity of colitis (R = 0.66, P = 0.04). The rWAS treatment also affected the MACB in wild type mice; specifically, microbial diversity was increased and the component ratios of *Proteobacteria* (*Phyllobacterium*) and *Deferribacteres* (*Mucispirillum*) were significantly decreased (S3 Fig).

**Table 3:** Detection ratio and mean abundance of operational taxonomic units (OTUs) changed in rWAS-exposed *Tcra*^-/-^ mice.

	OTU 4445673		OTU 184451		OTU 4481624		OTU 4409730	
	*Clostridium perfringens*		*Clostridium scindens*		*Clostridium disporicum*		*Clostridium bifermentans*	
	Detection ratio	Mean abundance	Detection ratio	Mean abundance	Detection ratio	Mean abundance	Detection ratio	Mean abundance
hetero (n = 5)	0%	n.d.	100%	0.5988%	80%	0.0181%	40%	0.0225%
control (n = 5)	0%	n.d.	100%	3.2644%	20%	0.0004%	20%	0.0313%
rWAS (n = 5)	60%	0.8404%	100%	0.1945%	60%	9.8127%	100%	0.7822%

n.d.; not detected.

hetero: *Tcra*^−/+^ mice not exposed to repeated water-avoidance stress (rWAS), control: *Tcra*^−/−^ mice not exposed to rWAS, rWAS: *Tcra*^−/−^ mice exposed to rWAS. Mean abundance represents the average of each sample in each group.

### Effect of LcS on chronic colitis exacerbated by rWAS

**Fig 3:**
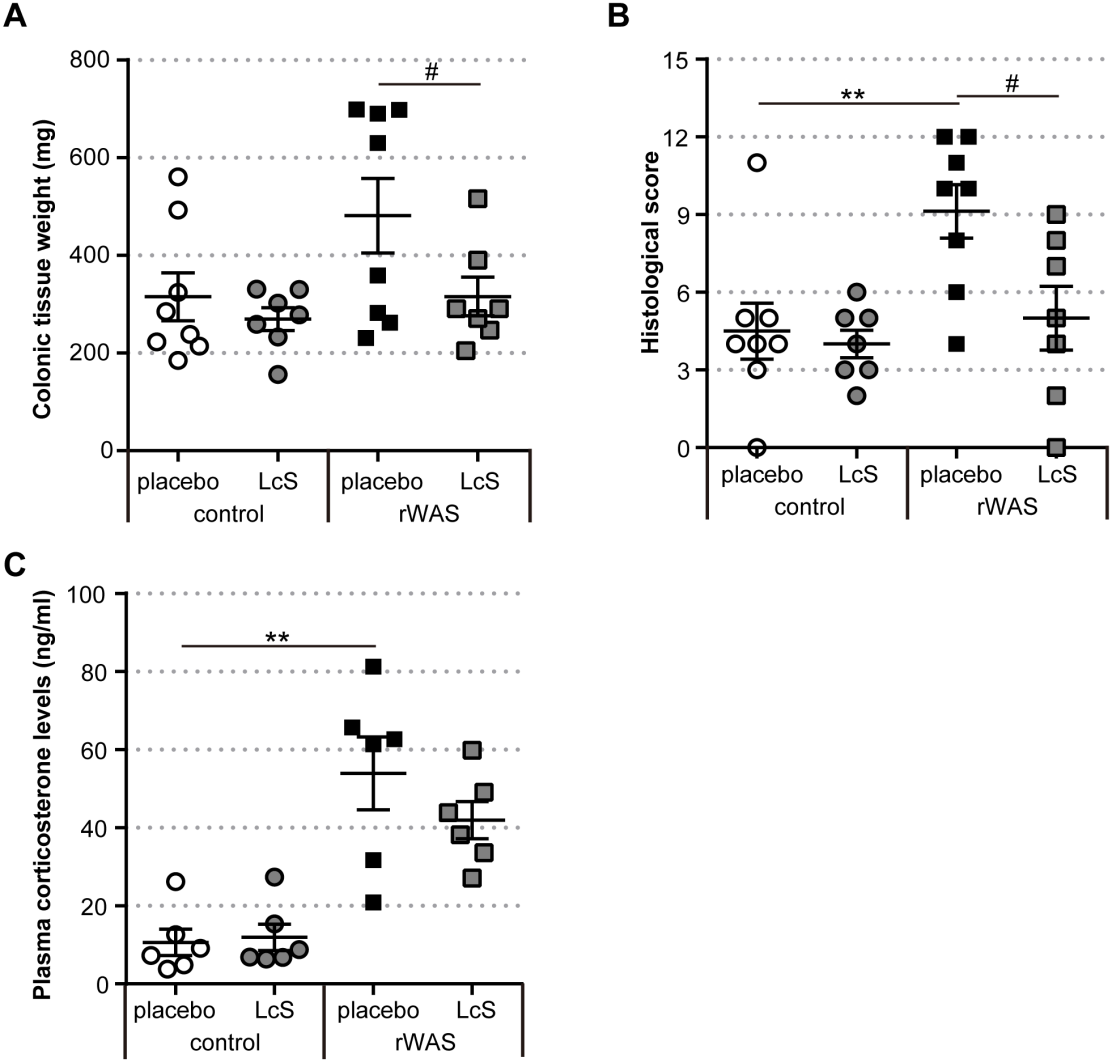
Effect of LcS on exacerbated colitis in *Tcra*^-/-^ mice exposed to rWAS. (A) Colonic tissue weight. (B) Histological score of colitis. Each point represents an individual animal; horizontal bar with errors represents mean ± SE, n = 7 ~ 8 per group. (C) Corticosterone concentration in the serum. Data are presented as mean ± SE, n = 6 per group. control: *Tcra*^−/−^ mice not exposed to repeated water avoidance stress (rWAS), rWAS: *Tcra*^−/−^ mice exposed to rWAS. Mice were given placebo or LcS-fermented milk, which were denoted as ‘placebo’ or ‘LcS’. * p < 0.05, ** p < 0.01 (control + placebo vs. rWAS + placebo), ^#^ p < 0.05 (rWAS + placebo vs. rWAS + LcS).

Next, we investigated the effect of ingested probiotic *Lactobacillus casei* strain Shirota (LcS) on chronic colitis exacerbated by exposing to rWAS in *Tcra*^-/-^ mice. rWAS exposure significantly increased disease activity, as estimated using colorectal tissue weight and histological score of proximal colon in placebo ingested *Tcra*^-/-^ mice (Fig 3A and 3B). On the other hand, LcS ingested *Tcra*^-/-^ mice during rWAS exposure resulted in suppression of the disease. The administration of LcS had no effect on the activation of the HPA axis caused by exposure to rWAS, as determined by estimating plasma corticosterone concentrations (Fig 3C). Moreover, the decline of disease in *Tcra*^-/-^ mice that ingested LcS during rWAS exposure was associated with inhibition of CD4^+^ TCRβ^dim^ T cell infiltration in the lamina propria (Table 4).

**Table 4:** Effect of LcS on LI-LPMC in *Tcra*^*-/-*^ mice during rWAS exposure.

	control		rWAS	
	placebo	LcS	placebo	LcS
Cell numbers (×10^4^ cells)				
CD4^+^ TCRβ^dim^	23.9 ± 13.0	11.9 ± 3.0	86.6 ± 42.5	13.0 ± 4.6
Gene expression levels of mRNA				
*UCN2*	0.6 ± 0.1	8.0 ± 1.6	12.9 ± 2.1*	5.9 ± 0.6^#^
*CRHR2*	0.6 ± 0.2	6.7 ± 1.3	13.1 ± 2.1*	6.1 ± 1.0^#^
*IFN-γ*	1.3 ± 0.5	2.7 ± 0.3	4.9 ± 1.0*	2.4 ± 0.6^#^
*IL-6*	1.8 ± 0.3	2.4 ± 0.4	4.0 ± 0.8	2.1 ± 0.1
*TNF-α*	1.4 ± 0.2	2.1 ± 0.1	2.2 ± 0.4	1.5 ± 0.2

control: *Tcra*^−/−^ mice not exposed to repeated water avoidance stress (rWAS), rWAS: *Tcra*^−/−^ mice exposed to rWAS. Mice were administrated placebo or LcS-fermented milk, which were denoted as ‘placebo’ or ‘LcS’. Data are expressed as mean ± SE, n = 6 per group.

* p < 0.05 (control + placebo vs. rWAS + placebo group),

^#^ p < 0.05 (rWAS + placebo vs. rWAS + LcS group).

The gene expression level of *IFN-γ* in LI-LPMC of rWAS-exposed and LcS treated *Tcra*^-/-^ mice was lower than those treated with placebo. Moreover, the expression of both *UCN2* and *CRHR2*, which increased with rWAS exposure, were repressed by LcS treatment in *Tcra*^-/-^ mice. Furthermore, LcS treatment also regulated the expression level of *IFN-γ*, *UCN2* and *CRHR2* increased by rWAS exposure in the normal condition mice (S2 Table).

### Effect of LcS on species diversity and disruption of MACB composition following rWAS exposure

**Fig 4:**
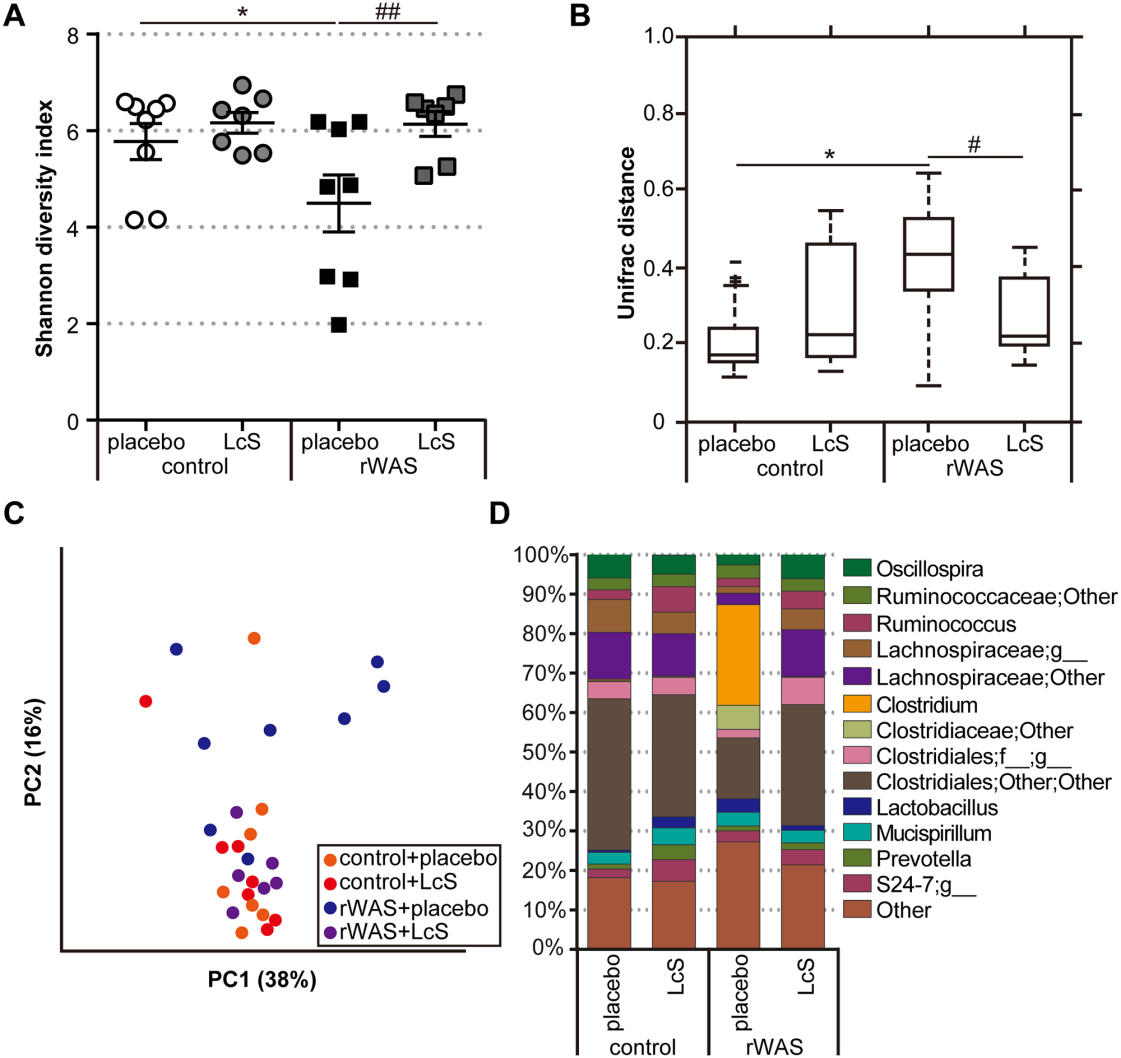
Effect of LcS on microbial diversity and composition in MACB of rWAS-exposed *Tcra*^-/-^ mice. (A) Shannon diversity index. Each point represents an individual animal; horizontal bar with errors represents mean ± SE, n = 7 ~ 8 per group. (B) Group distance. Data are presented as box plots, n = 7 ~ 8 per group. (C) Pcoa plot. Each point represents an individual animal, n = 7 ~ 8 per group. (D) Composition of MACB at the genus level. Data are presented as staked bar chart, n = 7 ~ 8 per group. control: *Tcra*^−/−^ mice not exposed to repeated water avoidance stress (rWAS), rWAS: *Tcra*^−/−^ mice exposed to rWAS. Mice are given placebo or LcS-fermented milk, which were denoted as the ‘placebo’ or ‘LcS’ group. * p < 0.05, *** p < 0.001 (control + placebo vs. rWAS + placebo), ^#^ p < 0.05, ^###^ p < 0.001 (rWAS + placebo vs. rWAS + LcS).

As shown in this study, exacerbation of colitis in rWAS-exposed *Tcra*^-/-^ mice was associated with loss of the microbial species richness as well as alterations in the genus composition of MACB. Moreover, we have shown that exacerbation of colitis in *Tcra*^-/-^ mice exposed to rWAS was inhibited by continuous ingestion of LcS during rWAS-exposure. Therefore, we next examined the effect of LcS ingestion on the microbial community of MACB in *Tcra*^-/-^ mice. We found that loss of species diversity in MACB of *Tcra*^-/-^ mice following rWAS exposure was inhibited by LcS treatment (Fig 4A). And the significant correlation between the species diversity and the severity of colitis was observed (R = -0.56, P < 0.01), which was well consistent with the data in Fig 2. The UniFrac distance in MACB of rWAS-exposed *Tcra*^*-/-*^ mice that received placebos was enlarged. However, LcS ingestion in *Tcra*^*-/-*^ mice during rWAS-exposure suppressed the increase in UniFrac distance (Fig 4B). The pcoa plot showed dispersed data points in rWAS-exposed *Tcra*^*-/-*^ mice that received placebos. On the other hand, when treated with LcS, the pcoa plot was clearly clustered, and was similar in position to the plot generated by control *Tcra*^-/-^ mice (Fig 4C). As expected, the component ratio of the genus *Clostridium* was significantly increased in MACB of rWAS-exposed *Tcra*^-/-^ mice that received placebos (Fig 4D). Analysis of the detection ratio and relative abundance of OTUs in the genus *Clostridium* showed that increased OTU 4445673 (*Clostridium perfringens*) and decreased OTU 184451 (*Clostridium scindens*) in the MACB of rWAS-exposed *Tcra*^-/-^ mice that received placebos (Table 5). However, those changes in MACB of rWAS-exposed *Tcra*^-/-^ mice were suppressed by LcS treatment. Notably, the component ratio of *Clostridium perfringens* was correlated with the severity of colitis (R = 0.50, P < 0.01). Similarly, LcS administration also inhibited the microbial changes in MACB of wild type mice, induced by rWAS exposure (S3 Fig).

**Table 5:** Effect of LcS on the OTUs significantly changed in MACB of rWAS-exposed *Tcra*^−/−^ mice.

	OTU 4445673		OTU 184451	
	*Clostridium perfringens*		*Clostridium scindens*	
	Detection ratio	Mean abundance	Detection ratio	Mean abundance
control				
placebo (n = 8)	75%	0.239%	88%	0.753%
LcS (n = 7)	29%	0.001%	86%	0.351%
rWAS				
placebo (n = 8)	100%	3.211%*	38%	0.008%*
LcS (n = 7)	100%	0.174%^#^	100%	2.774%^#^

control: *Tcra*^−/−^ mice not exposed to repeated water avoidance stress (rWAS), rWAS: *Tcra*^−/−^ mice exposed to rWAS. Mice were administrated placebo or LcS-fermented milk, which were denoted as ‘placebo’ or ‘LcS’. Mean abundance represents the average of each sample in each group.

* p < 0.05 (control + placebo vs. rWAS + placebo group),

^#^ p < 0.05 (rWAS + placebo vs. rWAS + LcS group).

### Effect of LcS on the rWAS-dependent increase in immunoglobulins in the luminal contents

**Fig 5:**
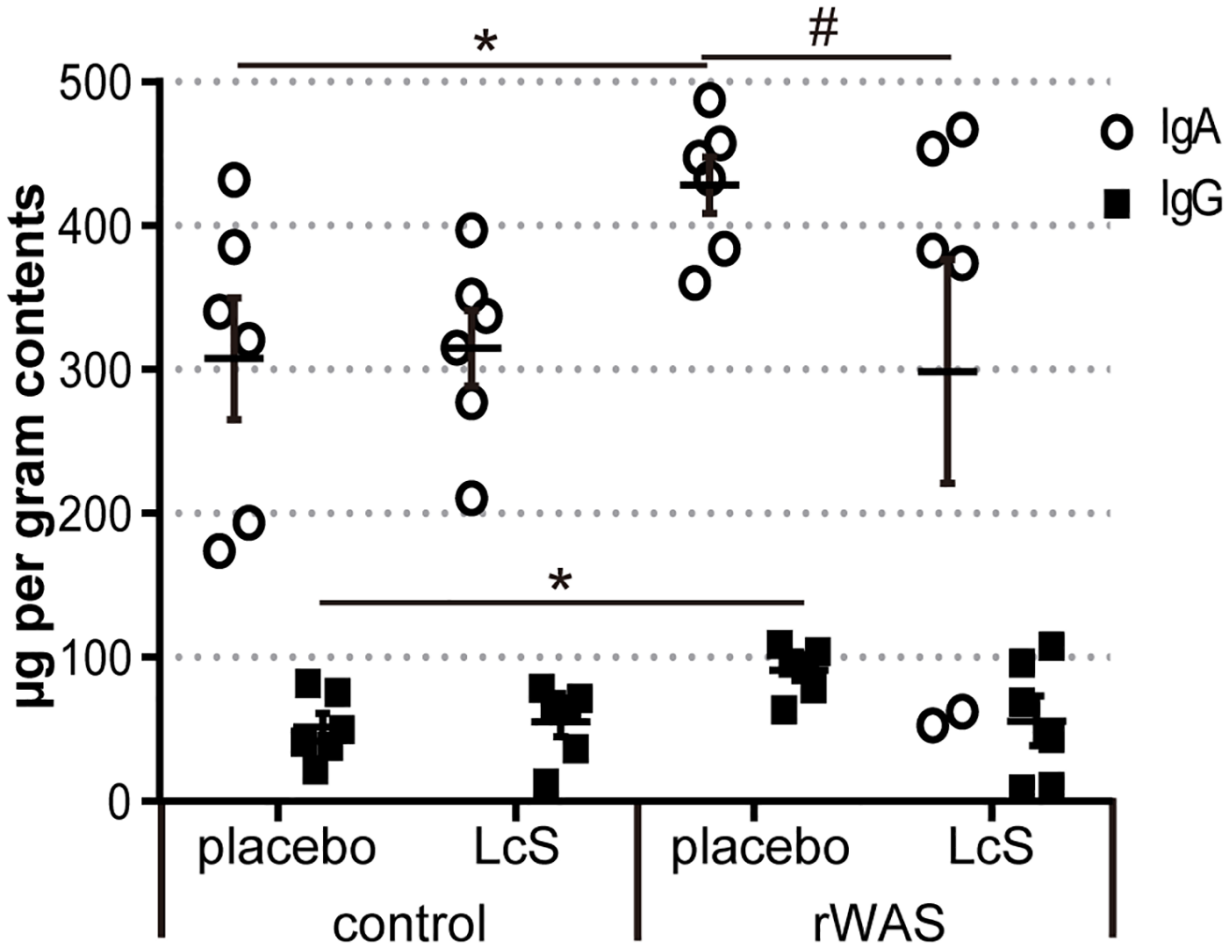
Effect of LcS on luminal IgA and IgG concentration in rWAS-exposed *Tcra*^-/-^ mice. Total IgA and IgG concentration in the colonic contents were measured by sandwich ELISA. control: *Tcra*^−/−^ mice not exposed to repeated water avoidance stress (rWAS), rWAS: *Tcra*^−/−^ mice exposed to rWAS. Mice were given placebo or LcS-fermented milk, which were denoted as ‘placebo’ or ‘LcS’ group. Data are represented as mean ± SE, n = 6 per group. * p < 0.05 (control + placebo vs. rWAS + placebo), ^#^ p < 0.05 (rWAS + placebo vs. rWAS + LcS).

We previously reported that the concentration of IgA and IgG in luminal contents of mice suffering from severe colitis was increased [25]. It was previously reported that certain gut microbiota are coated with IgG and IgA [36]. Moreover, secretory IgA with somatic hyper mutation (SHM) are essential for regulating the amount of GI microbiota [37]. It is possible that changes in the balance of secretory immunoglobulins resulted in disturbances in the MACB of *Tcra*^-/-^ mice during rWAS exposure. Concentrations of IgA and IgG in colonic contents of rWAS-exposed *Tcra*^-/-^ mice that received placebo were significantly higher than those in control *Tcra*^-/-^ mice. *Tcra*^*-/-*^ mice that ingested LcS during rWAS exposure showed similar IgG and IgA levels as compared with those of control mice (Fig 5).

## Discussion

To clarify the impact of chronic psychological stress on gut inflammation and microbial communities located on the colonic mucosal surface, we investigated the effect of 12-week rWAS treatment on B6-*Tcra*^*-/-*^ mice. We also investigated the probiotic effects of LcS on exacerbated colitis and its associated mucosal-flora dysbiosis during chronic psychological stress.

### Exacerbation of gut inflammation by chronic psychological stress

It was reported that *Tcra*^*-/-*^ mice spontaneously develop human UC-like colitis, which may be due to a deregulated mucosal immune system evoked by the GI microbiota [14, 38]. Previous studies indicated that unusual CD4^+^ T cell populations (CD4^+^ TCRβ^dim^ T cell) are responsible for the development of colitis in this strain of mice [39]. In this study, we confirmed that chronic exposure to psychological stress exacerbated colitis of *Tcra*^*-/-*^ mice, which was associated with severe CD4^+^ TCRβ^dim^ T cell infiltration in the colonic mucosa. Moreover, we confirmed that gene expression of IFN-γ was increased in LI-LPMC after rWAS exposure. In the case of acute colitis induced by DSS, short-term psychological stress has been shown to increase expression of IFN-γ, IL-6, and TNF-α, resulting in exacerbated colitis [18]. Therefore, we speculated that IFN-γ, which is secreted by LI-LPMC, is involved in exacerbation of colitis during continuous psychological stress. It is known that IFN-γ not only contributes to inflammatory responses in the gut, but also increases mucosal permeability via upregulation of myosin light chain kinase (MLCK), which regulates the expression of tight junction proteins [40]. An increase in mucosal permeability may cause alterations in the GI environment, and induce uptake of luminal antigens; these events are inferred to induce enhancement of the mucosal inflammatory response [41].

Psychological stress exerts effects on colorectal tissues via the neuroendocrine or central nervous system [22]. For the example, corticotropin-releasing hormone (CRH), which is secreted from hypothalamus in the HPA axis, is known to be involved in systemic stress responses such as enterokinesis, feeding regulation, and gastrointestinal motility [42, 43]. It was thought that CRH is predominantly expressed in the central nervous system, while urocortin (UCN) is expressed in the peripheral tissues [44]. In colorectal tissue, UCNs and CRHRs are expressed in mast cells, dendritic cells, and macrophages [44, 45], and the UCN-CRHR pathway is associated with both inflammatory and anti-inflammatory responses. For example, the UCN2-CRHR2 pathway was shown to induce colonic dysfunctions leading to irritable bowel syndrome (IBS) [46], and exacerbated colitis was induced by *Clostridium difficile* toxin A [47]. In contrast, CRHR2 signaling enhanced mucosal repair responses after DSS-induced colitis [48]. Our results demonstrated that *UCN2* and *CRHR2* gene expression was elevated by chronic psychological stress in not only inflammatory conditions but also physiological conditions in mice (S2 Table). Using a rWAS or maternal separation model, stress-induced CRHR2 signaling was shown to mediate colonic epithelial changes, including increased intestinal permeability [49], or acceleration of endotoxin tolerance, via overexpression of TLR4 and claudin-2 [50]. We hypothesized that UCNs are a marker of stress responses in the large intestine and that up-regulation of *UCNs* and *CRHRs* in LI-LPMC during chronic psychological stress is responsible for exacerbated mucosal inflammation.

### Relationship between changes in the MACB microbial community and exacerbation of gut inflammation induced by chronic psychological stress

Loss of bacterial species diversity of GI microbiota was previously observed in IBD patients and animal models [51, 52]. We analyzed the microbiota in the colonic contents of *Tcra*^*-/-*^ mice exposed to rWAS using 16S amplicon analysis and found drastic alterations in the composition of the colonic microbiota. Populations of endogenous pathogens such as *Clostridium perfringens* and *Clostridium sordellii* were expanded in rWAS-treated mice; both have been associated with exacerbation of colitis [25]. The present study focused on the changed microbial community in MACB during long-term exposure of psychological stress, as previous studies have suggested that GI microbiota are altered in patients with IBD [53]. We found loss of bacterial species diversity in MACB of *Tcra*^-/-^ mice exposed to chronic psychological stress. In addition, the significant increase in the component ratio of *Clostridium perfringens* and the significant decrease in that of *Clostridium scindens* were observed. These findings were consistent with previous reports outlining the changes in colonic contents of *Tcra*^*-/-*^ mice exposed to rWAS, except for the detection of *Clostridium* species, such as *Clostridium scindens* in MACB and *Clostridium sordellii* in colonic contents [25]. We speculated that these increased *Clostridium* species in colonic contents might have invaded into mucosal area. While the exact mechanisms contributing to such effects are unclear, it is possible that an increase of endogenous pathogens in MACB will affect mucosal barrier function, leading to accelerated colitis. For example, enterotoxins of *C*. *perfringens* were shown to interact with epithelial claudin family proteins, which induced dysregulation of epithelial tight-junctions, and increased the paracellular pathways [54]. Moreover, *Clostridium scindens* has been reported as a bile acid 7-dehydroxylating bacteria and is associated with microbiota-mediated resistance to *Clostridium difficile* infection [55]. Another study suggested that the mixture of 17 *Clostridia* strains, including *Clostridium scindens*, enhanced the differentiation of regulatory T cells (T_reg_) and attenuated colitis in a murine model [56]. It is possible that the decrease in the component ratio of *Clostridium scindens* is a factor contributing to the acceleration of colitis. Currently, the mechanisms involved in MACB disruption during rWAS exposure are still unclear. Previous reports have indicated that secretory IgA contributes to the close interaction between the gut microbiota and the host immune system; secretory IgA regulates diversity and composition of microbial communities in the gut, and well-balanced bacterial communities have been shown to induce Foxp3^+^ follicular T cells and differentiation of IgA-producing plasma cells [57]. Moreover, IgA- and IgG-coated GI microbiota were found to be increased in IBD, and IgA-coated microbiota contributed to the onset of colitis [36, 58]. Our analysis of the colonic contents in *Tcra*^*-/-*^ mice suggested that chronic psychological stress affected the differentiation of B cells and caused dysfunction of an IgA-mediated symbiotic regulatory loop between the host immune system and colonic microbiota [25]. In the present study, rWAS treatment in *Tcra*^-/-^ mice significantly increased the concentration of IgA in GI contents. Therefore, we speculate that changes in luminal IgA during rWAS induction may play key roles in the alteration of MACB microbial community.

### Effect of LcS on chronic psychological stress-induced gut inflammation

In the present study, we found that probiotic *Lactobacillus* strain (LcS) treatment clearly inhibited exacerbation of colitis induced by rWAS. However, LcS treatment did not inhibit colitis in control *Tcra*^-/-^ mice. The inhibitory effect of LcS in rWAS-exposed *Tcr*a^/-^ mice was associated with normalization of immunological and stress-induced parameters such as IFN-γ and UCN2/CRHR2 pathways in LI-LPMC. Moreover, LcS treatment stabilized microbial species diversity in MACB, which was significantly decreased by rWAS exposure. However, the differential effect of LcS on colitis between control and rWAS-treated mice remains unclear. It was previously described that Th2 cytokines such as IL-4 are involved in colitis induction in *Tcra*^-/-^ mice [59]. Meanwhile, we have found elevated *IFN-γ* in LI-LPMC of rWAS-exposed *Tcra*^-/-^ mice. It is possible that mechanisms differ between induction and acceleration of colitis by rWAS-exposure. Studies have shown that improvements following LcS treatment on DSS-induced chronic colitis were associated with a decrease in IFN-γ production and an increase in IL-4 production in LI-LPMC, when stimulated with CD3ε/CD28 [18]. Therefore, we hypothesized that LcS are effective for Th1-induced colitis, but not Th2-induced colitis. It is also possible that the improved effect of LcS on rWAS-induced deterioration of colitis was due to stabilization of MACB composition. As shown in this study, *Tcra*^-/-^ mice exposed to rWAS displayed a significant loss of microbial diversity. This phenotype was rescued when these mice ingested LcS. It was reported that loss of species richness and microbial diversity are also present in patients with IBD [14]. Recent reports indicated that fecal microbiota transplantation (FMT) in patients with *Clostridium difficile* infection (CDI) demonstrated clinical effectiveness, and is associated with recovery of species richness and microbial diversity in CDI patients [60]. As described above, loss of species richness and microbial diversity are observed not only in CDI patients but also in IBD patients. Therefore, it is possible that restoration of the microbial species diversity in MACB via FMT can be used as a new therapeutic strategy in exacerbated IBD. Ingestion of probiotic treatments in IBD patients will be one of these strategies for MACB targeted therapies, as probiotics are known to have beneficial effects on the maintenance of gut microbiota balance [61]. In this study, we have confirmed the effectiveness of LcS treatment on rWAS-exposed *Tcra*^-/-^ mice, and showed that it is beneficial for maintaining microbial diversity in MACB in addition to inhibiting the infiltration of several *Clostridium* species. It should be noted that the level of luminal immunoglobulins in rWAS-afflicted *Tcra*^-/-^ mice supplied with LcS was similar to that of control *Tcra*^-/-^ mice, while those given the placebos demonstrated an increase in luminal immunoglobulin levels. It is well established that some probiotic strains exhibit adjuvanticity by enhancing antigen-specific IgA production [62, 63]. Moreover, studies have shown that one strain of *Lactobacillus gasseri* can induce IgA production and increase the IgA^+^ cell population in both Peyer’s patch and lamina propria [64]. Collectively, modulation of immunoglobulin synthesis by LcS in the gut may play a putative role in the regulation of microbial communities in MACB.

## Conclusion

In this study, we elucidated the effect of chronic psychological stress on the composition of mucosa-associated bacterial flora (so called as MACB) and mucosal immune responses. We have shown that chronic psychological stress mediates recurrence of active IBD using an animal model of UC. *Tcra*^-/-^ mice exposed to rWAS exhibited accelerated chronic colitis, which was associated with elevated inflammatory cytokines and stress-mediated peptide mRNA expression in LI-LPMC. In addition, rWAS-exposure induced loss of the microbial diversity in MACB, and increased luminal concentrations of immunoglobulins. These results led us to hypothesize that MACB may function as one of the mucosal barriers. Further studies should be focused on determining the physiological roles of MACB in intestinal homeostasis. Another important finding in this study was that treatment of probiotic lactobacillus strain inhibited acceleration of colitis in rWAS-exposed *Tcra*^-/-^ mice, and stabilized microbial species diversity in MACB. Taken together, our results suggest that maintenance of microbial diversity in MACB will be important in designing therapeutic targets for the treatment of IBD.

## Supporting Information

S1 Fig Effects of rWAS frequency on the exacerbation of colitis and serum corticosterone concentration in *Tcra*^−/−^ mice. (A) Colonic tissue weight. (B) Histological scores of colitis. Each point represents an individual animal; horizontal bar with errors represents mean ± SE, n = 12 per group. (C) Corticosterone concentration in the serum. Data are presented as mean ± SE, n = 5 per group. control: *Tcra*^−/−^ mice not exposed to repeated water avoidance stress (rWAS), lf-rWAS: *Tcra*^−/−^ mice exposed to rWAS (once a week for 12 weeks), rWAS: *Tcra*^−/−^ mice exposed to rWAS (five times per week for 12 weeks). * p < 0.05, ** p < 0.01 (vs. control), ^#^ p < 0.05 (lf-rWAS vs. rWAS).
(TIF)

S2 Fig Effects of rWAS and administration of LcS on colonic tissue weight and corticosterone concentration in the serum of C57BL/6 mice. (A) Colonic tissue weight. Each point represents an individual animal; horizontal bar with errors represents mean ± SE, n = 5 per group. (B) Corticosterone concentration in the serum. Data are represented as mean ± SE, n = 5 per group. (C) Scores of fecal characteristics. Score was estimated as 0–3 (0: strict, 1: smooth and soft, 2: fluffy, 3; watery). Each point represents an individual animal; horizontal bar with errors represents mean ± SE, n = 5 per group. control: C57BL/6 mice not exposed to rWAS, rWAS: C57BL/6 mice exposed to rWAS. Mice exposed to rWAS were given placebo or LcS-fermented milk, which were denoted as ‘placebo’ or ‘LcS’ group. * p < 0.05 (control vs. rWAS + placebo), ^#^ p < 0.05 (rWAS + placebo vs. rWAS + LcS).
(TIF)

S3 Fig Effects of rWAS and administration of LcS on microbial species diversity and genus composition in MACB of C57BL/6 mice. (A) Shannon diversity index. Each point represents an individual animal; horizontal bar with errors represents mean ± SE, n = 5 per group. (B) Composition of MACB in the large intestine at the genus level. Data are presented as staked bar chart, n = 5 per group. control: C57BL/6 mice not exposed to rWAS, rWAS: C57BL/6 mice exposed to rWAS. Mice exposed to rWAS were given placebo or LcS-fermented milk, which were denoted as ‘placebo’ or ‘LcS’ group. ** p < 0.01 (control vs. rWAS + placebo), ^#^ p < 0.05 (rWAS + placebo vs. rWAS + LcS).
(TIF)

S1 Materials and Methods (DOCX)

S1 Table Effect of rWAS on the activity of myeloperoxidase in colonic contents and mRNA expression in LI-LPMC of *Tcra*^*-/-*^ mice. n.d.: not detected. Activity of myeloperoxidase was measured by the enzyme assay. Gene expression of mRNA in LI-LPMC was determined by quantitative RT-PCR with ABI-7500. control: *Tcra*^−/−^ mice not exposed to repeated water avoidance stress (rWAS), rWAS: *Tcra*^−/−^ mice exposed to rWAS. Data are presented as mean ± SE, n = 5 per group.
(DOCX)

S2 Table Effects of rWAS treatment and administration of LcS on mRNA expression in LI-LPMC of C57BL/6 mice. Gene expression of mRNA in LI-LPMC was determined by quantitative RT-PCR with an ABI-7500. control: C57BL/6 mice not exposed to repeated water avoidance stress (rWAS), rWAS: C57BL/6 mice exposed to rWAS. Mice exposed to rWAS were given placebo or LcS-fermented milk, which were denoted as ‘placebo’ or ‘LcS’ group. Data are presented as mean ± SE, n = 5 per group. * p < 0.05, ** p < 0.01, *** p < 0.001 (control vs. rWAS + placebo), ^##^ p < 0.01, ^###^ p < 0.001 (rWAS + placebo vs. rWAS + LcS).
(DOCX)
