# Supplementary figures and images for: Disturbance in the Mucosa-Associated Commensal Bacteria Is Associated with the Exacerbation of Chronic Colitis by Repeated Psychological Stress; Is That the New Target of Probiotics?
Source: PLoS One. 2016 Aug 8;11(8):e0160736. doi: 10.1371/journal.pone.0160736 (PMC4976886; doi:10.1371/journal.pone.0160736)

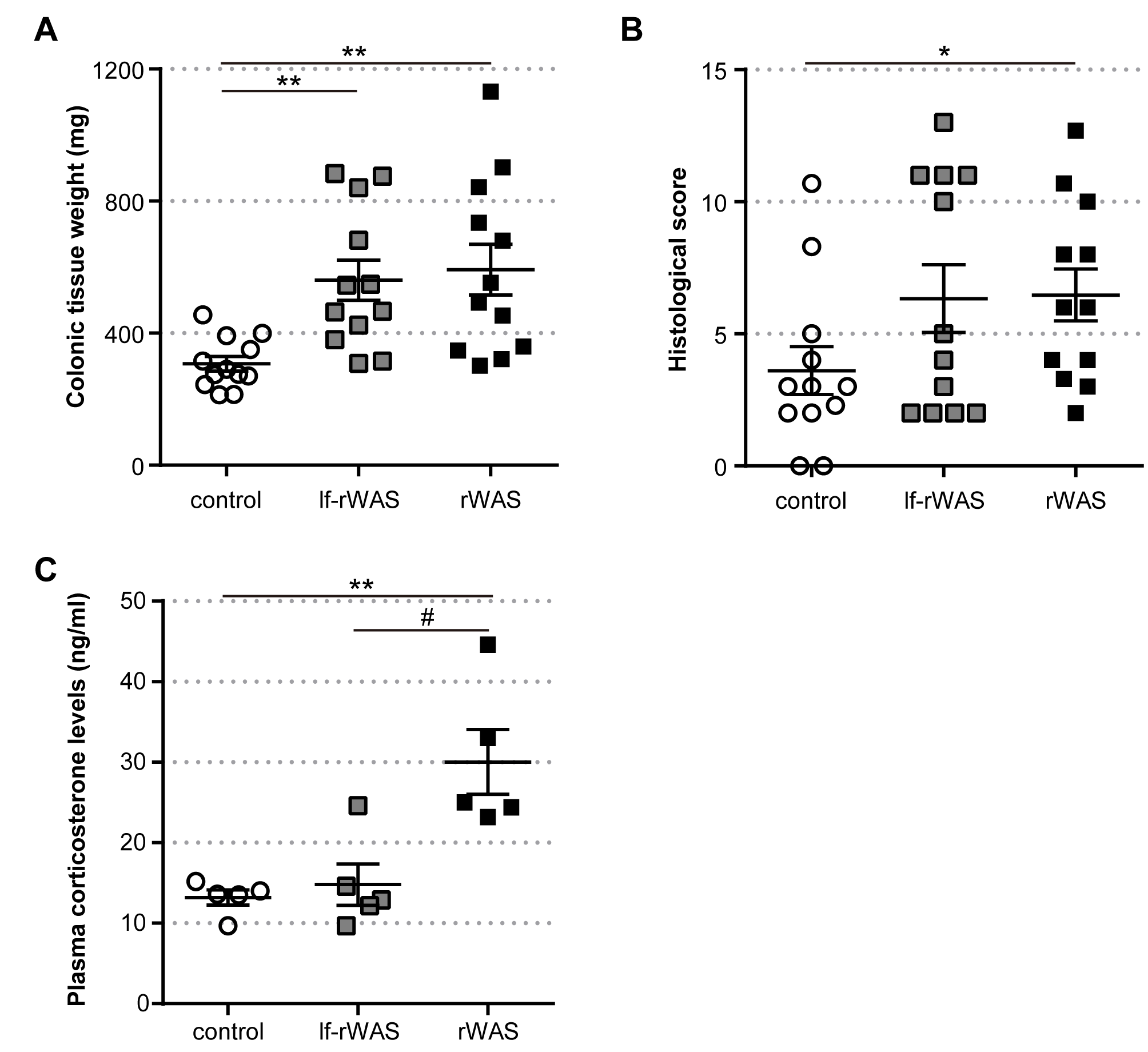

Supplement: S1 Fig — (A) Colonic tissue weight. (B) Histological scores of colitis. Each point represents an individual animal; horizontal bar with errors represents mean ± SE, n = 12 per group. (C) Corticosterone concentration in the serum. Data are presented as mean ± SE, n = 5 per group. control: Tcra−/− mice not exposed to repeated water avoidance stress (rWAS), lf-rWAS: Tcra−/− mice exposed to rWAS (once a week for 12 weeks), rWAS: Tcra−/− mice exposed to rWAS (five times per week for 12 weeks). * p < 0.05, ** p < 0.01 (vs. control), # p < 0.05 (lf-rWAS vs. rWAS). (TIF) [file pone.0160736.s001.tif]

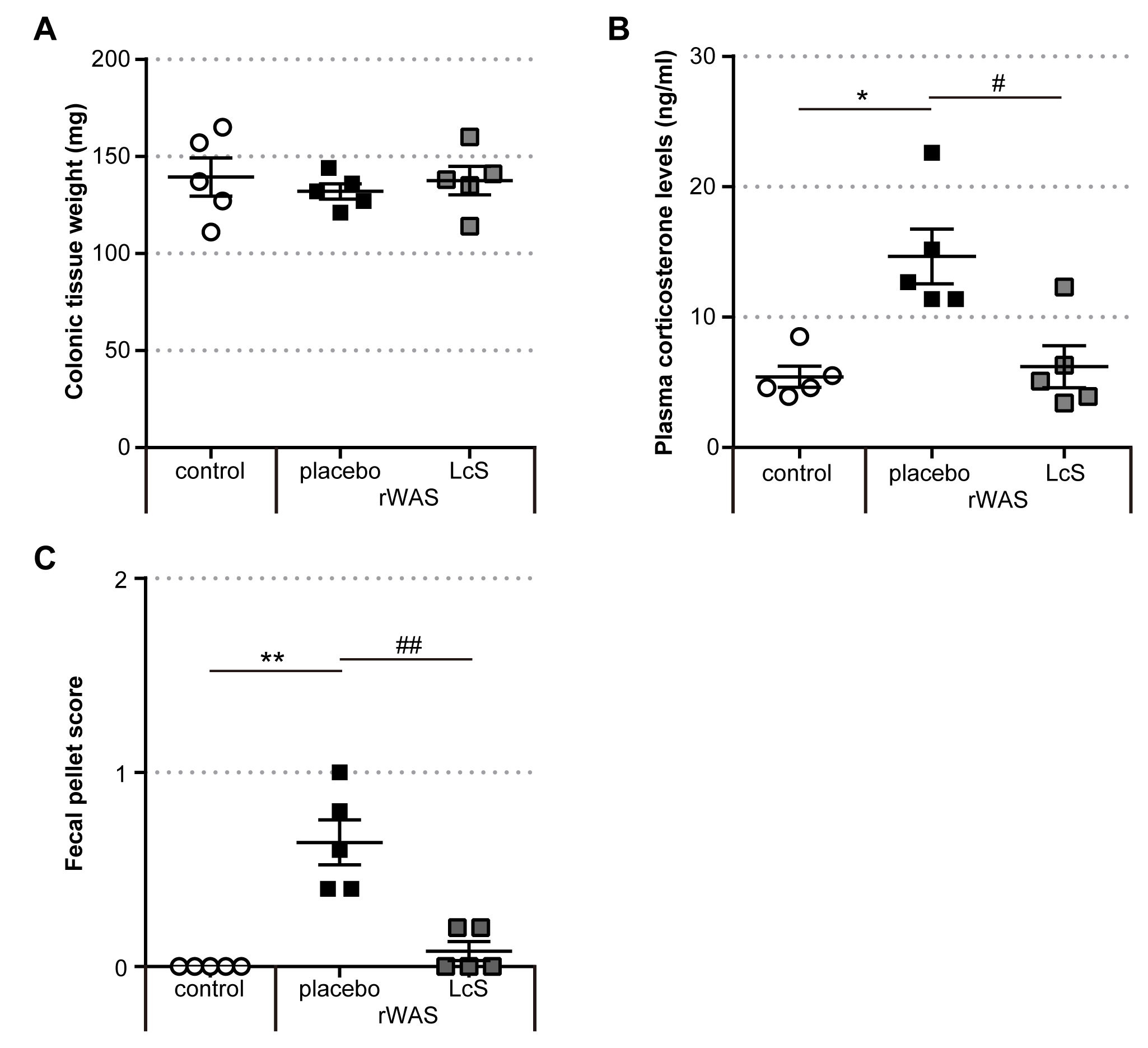

Supplement: S2 Fig — (A) Colonic tissue weight. Each point represents an individual animal; horizontal bar with errors represents mean ± SE, n = 5 per group. (B) Corticosterone concentration in the serum. Data are represented as mean ± SE, n = 5 per group. (C) Scores of fecal characteristics. Score was estimated as 0–3 (0: strict, 1: smooth and soft, 2: fluffy, 3; watery). Each point represents an individual animal; horizontal bar with errors represents mean ± SE, n = 5 per group. control: C57BL/6 mice not exposed to rWAS, rWAS: C57BL/6 mice exposed to rWAS. Mice exposed to rWAS were given placebo or LcS-fermented milk, which were denoted as ‘placebo’ or ‘LcS’ group. * p < 0.05 (control vs. rWAS + placebo), # p < 0.05 (rWAS + placebo vs. rWAS + LcS). (TIF) [file pone.0160736.s002.tif]

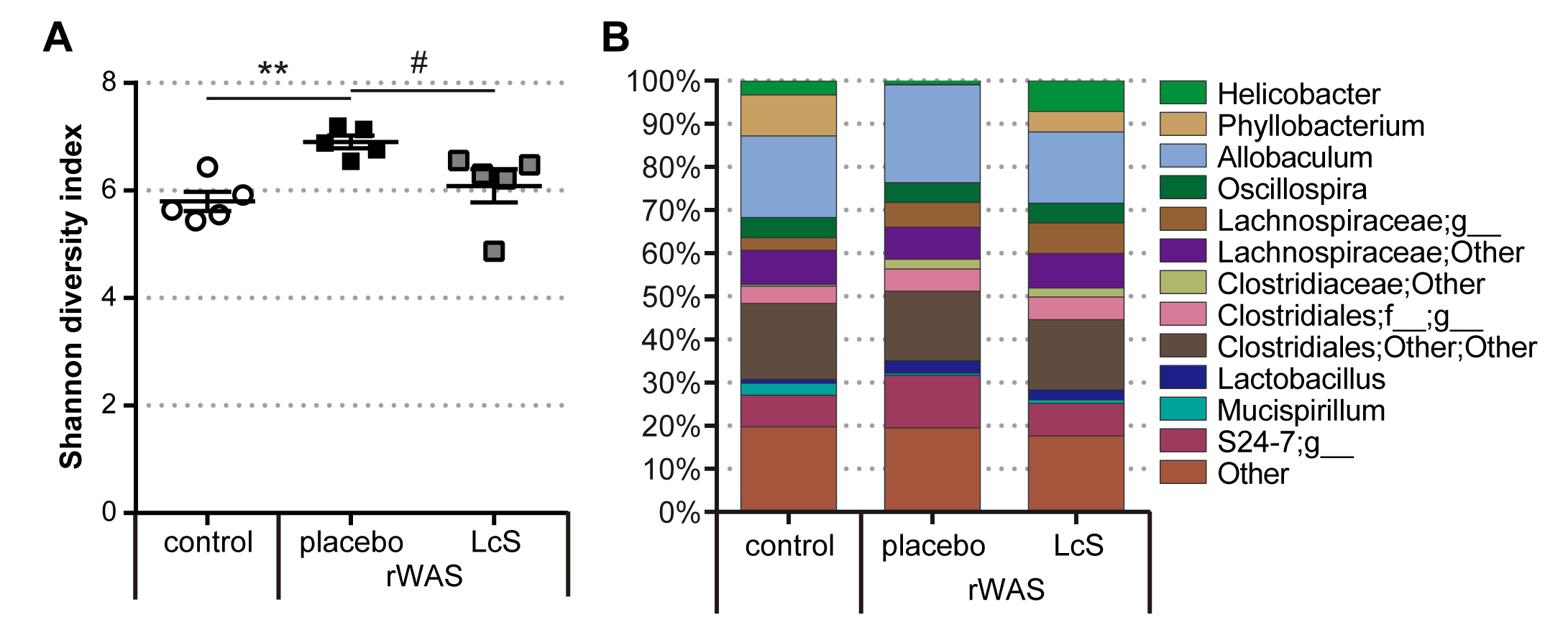

Supplement: S3 Fig — (A) Shannon diversity index. Each point represents an individual animal; horizontal bar with errors represents mean ± SE, n = 5 per group. (B) Composition of MACB in the large intestine at the genus level. Data are presented as staked bar chart, n = 5 per group. control: C57BL/6 mice not exposed to rWAS, rWAS: C57BL/6 mice exposed to rWAS. Mice exposed to rWAS were given placebo or LcS-fermented milk, which were denoted as ‘placebo’ or ‘LcS’ group. ** p < 0.01 (control vs. rWAS + placebo), # p < 0.05 (rWAS + placebo vs. rWAS + LcS). (TIF) [file pone.0160736.s003.tif]
