# Supplementary material for: Disturbance in the Mucosa-Associated Commensal Bacteria Is Associated with the Exacerbation of Chronic Colitis by Repeated Psychological Stress; Is That the New Target of Probiotics?
Source: PLoS One. 2016 Aug 8;11(8):e0160736. doi: 10.1371/journal.pone.0160736 (PMC4976886; doi:10.1371/journal.pone.0160736)
Supplement: S1 Materials and Methods — (DOCX) [file pone.0160736.s004.docx]

**Materials and methods**

**Mice**

Female 8 week old C57BL/6 mice were purchased (CLEA Japan, Inc., Tokyo, Japan). Animals were maintained in ventilated cages containing autoclaved soft bedding, water, and food, in an environmentally controlled room (23 ± 1°C, 12 h light/dark cycle). After the stress session, mice were euthanized by isoflurane inhalation and the samples were collected for analysis. All animal experiments were approved by the Institutional Animal Care and Use Committee of Yakult Central Institute (approval number: 14-0257).

The rWAS exposure method and all analysis in C57BL/6 mice were performed as stated for *Tcra^-/-^* mice, in the manuscript.
