# Supplementary material for: Disturbance in the Mucosa-Associated Commensal Bacteria Is Associated with the Exacerbation of Chronic Colitis by Repeated Psychological Stress; Is That the New Target of Probiotics?
Source: PLoS One. 2016 Aug 8;11(8):e0160736. doi: 10.1371/journal.pone.0160736 (PMC4976886; doi:10.1371/journal.pone.0160736)
Supplement: S1 Table — n.d.: not detected. Activity of myeloperoxidase was measured by the enzyme assay. Gene expression of mRNA in LI-LPMC was determined by quantitative RT-PCR with ABI-7500. control: Tcra−/− mice not exposed to repeated water avoidance stress (rWAS), rWAS: Tcra−/− mice exposed to rWAS. Data are presented as mean ± SE, n = 5 per group. (DOCX) [file pone.0160736.s005.docx]

**S1 Table. Effect of rWAS on the activity of myeloperoxidase in colonic contents and mRNA expression in LI-LPMC of *Tcra^-/-^* mice.**

|  | control | rWAS |
| --- | --- | --- |
| Activity of  myeloperoxidase  (U/ml/min) | 0.12 ± 0.03 | 0.39 ± 0.10 |
| Gene expression levels of mRNA | | |
| *IL-1β* | 0.9 ± 0.3 | 1.4 ± 0.6 |
| *IL-10* | 1.1 ± 0.2 | 0.6 ± 0.2 |
| *IL-17α* | 0.9 ± 0.2 | 1.2 ± 0.6 |
| *IL-18* | 1.1 ± 0.2 | 0.8 ± 0.2 |
| *IL-22* | 0.8 ± 0.2 | 0.5 ± 0.1 |
| *IL-23* | n.d. | n.d. |

n.d.: not detected. Activity of myeloperoxidase was measured by the enzyme assay. Gene expression of mRNA in LI-LPMC was determined by quantitative RT-PCR with ABI-7500. control: *Tcra*^−/−^ mice not exposed to repeated water avoidance stress (rWAS), rWAS: *Tcra*^−/−^ mice exposed to rWAS. Data are presented as mean ± SE, n = 5 per group.
