# Supplementary material for: Disturbance in the Mucosa-Associated Commensal Bacteria Is Associated with the Exacerbation of Chronic Colitis by Repeated Psychological Stress; Is That the New Target of Probiotics?
Source: PLoS One. 2016 Aug 8;11(8):e0160736. doi: 10.1371/journal.pone.0160736 (PMC4976886; doi:10.1371/journal.pone.0160736)
Supplement: S2 Table — Gene expression of mRNA in LI-LPMC was determined by quantitative RT-PCR with an ABI-7500. control: C57BL/6 mice not exposed to repeated water avoidance stress (rWAS), rWAS: C57BL/6 mice exposed to rWAS. Mice exposed to rWAS were given placebo or LcS-fermented milk, which were denoted as ‘placebo’ or ‘LcS’ group. Data are presented as mean ± SE, n = 5 per group. * p < 0.05, ** p < 0.01, *** p < 0.001 (control vs. rWAS + placebo), ## p < 0.01, ### p < 0.001 (rWAS + placebo vs. rWAS + LcS). (DOCX) [file pone.0160736.s006.docx]

**S2 Table. Effects of rWAS treatment and administration of LcS on mRNA expression in LI-LPMC of C57BL/6 mice.**

|  | control | rWAS | |
| --- | --- | --- | --- |
|  |  | placebo | LcS |
| *UCN2* | 0.9 ± 0.1 | 2.7 ± 0.3*** | 1.0 ± 0.1^##^ |
| *CRHR2* | 0.9 ± 0.2 | 3.9 ± 0.2** | 1.3 ± 0.2^##^ |
| *IFN-γ* | 1.1 ± 0.2 | 3.1 ± 0.2* | 1.2 ± 0.2^###^ |

Gene expression of mRNA in LI-LPMC was determined by quantitative RT-PCR with an ABI-7500. control: C57BL/6 mice not exposed to repeated water avoidance stress (rWAS), rWAS: C57BL/6 mice exposed to rWAS. Mice exposed to rWAS were given placebo or LcS-fermented milk, which were denoted as ‘placebo’ or ‘LcS’ group. Data are presented as mean ± SE, n = 5 per group. * p < 0.05, ** p < 0.01, *** p < 0.001 (control vs. rWAS + placebo), ^##^ p < 0.01, ^###^ p < 0.001 (rWAS + placebo vs. rWAS + LcS).
